# Supplementary material for: Annona muricata L.-Derived Polysaccharides as a Potential Adjuvant to a Dendritic Cell-Based Vaccine in a Thymoma-Bearing Model
Source: Nutrients. 2020 May 29;12(6):1602. doi: 10.3390/nu12061602 (PMC7352220; doi:10.3390/nu12061602)
Supplement: Supplementary file 1 [file nutrients-12-01602-s001.zip › Figure S1.docx]

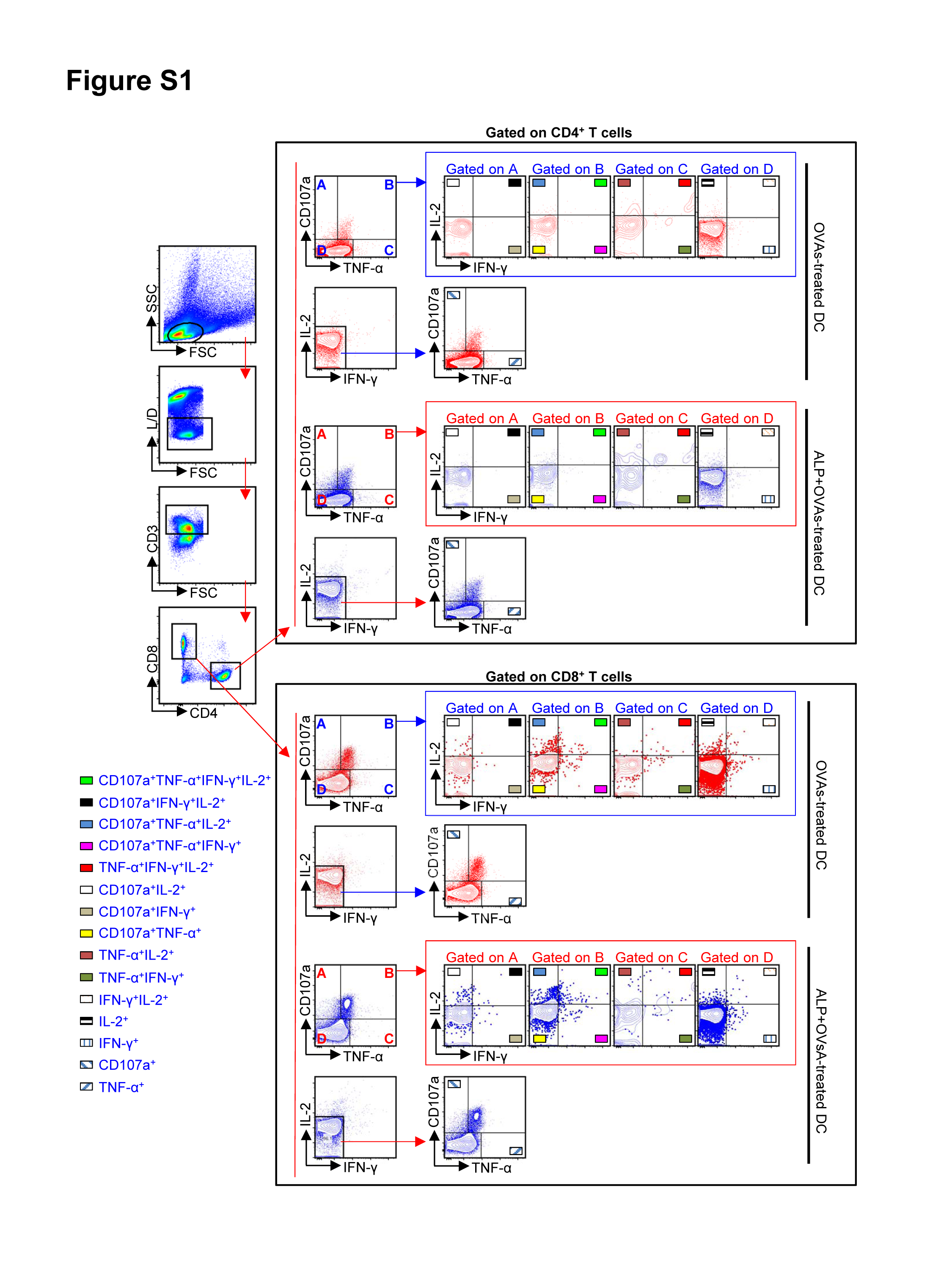


**Figure S1.** Flow cytometry analysis of multifunctional T cells. Spleen cell suspensions were analyzed using multiparameter flow cytometry for CD107a and cytokines production, and data were collected on a FACSverse flow cytometer with subsequent analysis using FlowJo software. An inclusion gate was first drawn based on the cell size and granularity to analyze the cytotoxic cell marker (CD107a) and cytokine-producing T cells (IFN-γ, IL-2, or TNF-α). Subsequently, the dead cell population was excluded using the Live/Dead, and the CD3^+^ lymphocytes were gated. T cells were further gated to obtain CD4^+^ and CD8^+^ T cells. Gates indicating positive staining for each CD107a and cytokines in 2 immunized groups (OVAs-DC *vs* ALP/OVAs-DC) were delineated using the non-treated T cells to distinguish multifunctional T cell subsets.
